# Self-compassion, Positive and Negative Affect and Social Avoidance among Adolescence: Mediating Role of Mindfulness

**DOI:** 10.2174/0117450179268979231114045116

**Published:** 2023-11-17

**Authors:** M.S. Sujamani, Barani Kanth

**Affiliations:** 1 Department of Psychology, Presidency College, Chennai, India; 2 Department of Applied Psychology, Pondicherry University, Pondicherry-605014, India

**Keywords:** Self-compassion, Mindfulness, Social anxiety, Adolescence, Positive affect, Negative affect

## Abstract

**Objective:**

Mindfulness is an attribute of consciousness to manage social fear avoidance and promote well-being. Social anxiety is a common psychological experience noted among the general population. Social anxiety develops during adolescence and is prevalent among college students. This study investigates the factors contributing to social anxiety - fear and avoidance of social situations of female first-year undergraduates.

**Methods:**

The study used a survey research design. A sample of 821 first-year female undergraduate students aged between 17 and 19. Data were collected using the Liebowitz Social anxiety scale, the Five-Facet mindfulness questionnaire, A short form of the Self-compassion scale, and the Positive and Negative affect scale.

**Results:**

Mindfulness weakens social fear and reduces the tendency to avoid social situations. Mindfulness effectively mediates the impact of self-compassion's positive affect and negative effects on social fear. Mindfulness and social fear jointly mediate the impact of self-compassion, positive affect, and negative affect on social avoidance.

**Conclusion:**

Mindfulness is the awareness and acceptance of the feelings, thoughts and sensations attached to self and its possible reciprocity with social surroundings to mitigate fear—self-compassion and positive emotional affect augment, and negative emotional affect attenuate mindfulness. Results analysis highlights the mediation of mindfulness on social anxiety, self-compassion, positive affect, and negative affect.

## 1 INTRODUCTION

Social anxiety (SA) is an outcome of fear of evaluation by others in situations demanding social interaction, like discussing in groups, facing interviews, interacting with strangers, speaking in public, dating, *etc*. When this fear becomes severe and debilitating, it is referred to as social anxiety disorder (SAD), a clinical condition [1]. Both correlational and experimental studies have aptly demonstrated the role of cognitive, affective, and behavioral factors in the onset and maintenance of SA and SAD. According to the cognitive-behavioral model, the fear of evaluation originates from a strong desire on the part of individuals to make favorable impressions on others, who, in their estimation, are likely to expect high social performance standards and evaluate them against those standards. Expectations of high-performance standards by others in social situations restrict people from focusing unduly on the threat-provoking aspects of social interactions [2] and shifting attention away from positive social information [3]. Such positive social information is often threatening [4] because it raises performance standards in subsequent social interactions and makes them conspicuous in social situations [5, 6], which is often intimidating. Consequently, individuals with high SA tend to develop negative emotional and cognitive memories and imageries of social interactions [7], which become internalized and undermine their self-image. As a result, they are less likely to associate self with positive social attributes [8, 9].

During social interactions, the attention of individuals with high SA becomes excessively self-focused, with an acute awareness of bodily sensations, feelings, thoughts, and memories [10]. Emotional control gets out of hand, leading to difficulty regulating emotional experiences and expressions [11, 12]. The poor self-appraisal coupled with negative emotional experience engender feelings of inadequacy and inferiority, resulting in embarrassment and humiliation while dealing with social interactions. In such situations, individuals with high SA either avoid social events or indulge in excessive self-monitoring and rehearsal to impress others [13, 14]. Using self-protective behaviors elevates anxiety levels, negative outcomes of social events, and poorer performance ratings by self and others [15, 16]. These negative self-experiences are more likely to result in less positive post-event processing [17-20]. In this way, SA is perpetuated by forming negative self-impressions and retrieving negative memories following a social event or anticipating an upcoming social event [21]. This vicious cycle denies individuals the opportunity to reality check and develop adequate social competencies [22, 24].

The role of negative self-evaluation and emotional affect in the onset and maintenance of SA is amply demonstrated in clinical and non-clinical populations. The negative self-evaluations arising from negative experiences encountered in social situations [25] and consequent faulty beliefs about one's competency in dealing with social interactions [22] undermine an individual's ability to cope with social situations. Previous studies have shown that due to strict social rules of behavior and a lack of adequate social competencies, young adults in India go through embarrassment, guilt, self-blame, significant gender inequality and insensitivity, unfair social treatment, and deprivation [23, 24]. Avenues for professional guidance and training in developing social competencies are also very limited, given that they are non-clinical, and young adults dislike the social stigma attached to seeking professional help. Hence, understanding the dynamics of SA in this group is necessary for helping this group overcome this undesirable psychological predisposition.

Self-compassion is characterized by kindness towards one's pains and misfortunes, a feeling of connectivity with common humanity as failures and mistakes are common to all humans, and being mindful of one's pains and misfortunes by being aware and accepting [26]. Mata analytic studies have shown significant negative relationships between self-compassion, a personal predisposition characterized by open-minded, realistic, and compassionate self-evaluations, and several psychopathological conditions [27]. High self-compassion is associated with a reduced likelihood of ruminations about past feelings or being overwhelmed by feelings of inadequacy [26], engaging less in debilitating self-focused attention, thereby enabling attentional focus on the external environment [28, 29] and showing reduced reliance on cognitive and behavioral avoidance strategies to facilitating realistic appraisal of social situations [15, 30].

## 2 CONCEPTUAL FRAMEWORK OF THE STUDY

The overt manifestation of SA is the avoidance of social situations. Covert feelings of fear of social situations propel this behavioral avoidance. Fear in social situations leads to actively avoiding them as an irrational defense. Hence, to address SA, the social fear must be dealt with. Once social fear is mitigated, attempts to approach social situations strengthen, giving rise to opportunities for learning appropriate social skills. Based on this premise, this study has been conceived to understand the antecedent conditions that impact SA (fear and avoidance). Onset and maintenance of SA are found to be closely associated with self-compassion [25, 31-37]. These factors may not directly impact SA but create awareness, understanding, and acceptance of one's feelings, thoughts, sensations, and outer reality with all its ramifications. This mindful attitude towards self and surroundings may act as a preparatory set and could unleash coping mechanisms to deal with and attenuate the fear of social situations. Reduced social fear may help the realistic appraisal of social situations, which may guard against the temptation to get overwhelmed and flee the social situations but facilitate approach behavior, providing ample opportunities for learning social skills.

### 2.1 The Conceptual Model

Fig. (**1**) depicts that self-compassion (SC) and positive emotional affect (PA) will facilitate, and negative emotional affect (NA) will hinder one's awareness and acceptance of self and social situations (MD). This awareness and acceptance of self and the environment will mitigate or aggravate the fear of social situations (SFR). The reduced or increased social fear, in turn, will reduce or increase social avoidance (SAV), leading to either participation in or avoidance of social events, thus increasing or diminishing the opportunities for learning social skills. Mindfulness and social fear will mediate both individually and jointly the relationship impact of self-compassion and the positive and negative emotional effects of social avoidance.

The most dominant cognitive factor is attention bias and reduced attention control. People with SA find it difficult to process positive social information [3] and cannot shift their attention away from threat-provoking social information [2]. They interpret positive social interactions as threatening [4], often fail to accept others' positive reactions to social events [31], and endorse more negative interpretations of positive events [32]. This happens mainly due to the fear that higher standards of social performance would be expected of them by others. Inwardly, they are less likely to have implicit associations between self and positive social attributes [8, 9] and tend to develop negative emotional and cognitive visual memories of recent social interactions. The Positive imagery is relatively impoverished and degraded episodic details [7]. Their attention is self-focused, with an acute awareness of bodily sensations, feelings, thoughts, and memories, which further increase their anxiety levels [10].

Emotional regulation in individuals with SA appears to be imperfect. They tend to pay scanty attention to their emotional experiences and their descriptions of the experiences more ambiguously [12]. They suppress emotions and exhibit greater ambivalence in emotional expression due to the fear that it may lead to social rejection [11]. Their response to positive information and experiencing positive emotions are particularly impaired [5]. They exhibit a fear of positive evaluation to avoid being conspicuous and circumvent raising standards by which they will be evaluated in the future [6]. Fear of positive emotions compels them to dampen positive affect in savoring and expressing and maintain low levels of positive affect [33, 12]. They report higher levels of anger but suppress anger expression and direct it inward [34].

Dysfunctional behavior patterns are displayed to prevent feared social outcomes. These self-protective strategies do not help alleviate anxiety but only maintain it [14]. They can be classified into avoidance (low self-disclosure, avoidance of eye contact, attempts to conceal anxiety) and impression management (excessive self-monitoring and rehearsal) subtypes [13]. The use of safety behaviors only resulted in higher levels of anxiety, negative predictions about the outcome of social events, and poorer ratings of performance by self and others [15]. At the same time, reduced use of safety behaviors reduced negative predictions about social outcomes and increased positive ratings of participant's social behavior by others [16]. SA is perpetuated by forming negative self-impressions and retrieving negative memories following a social event or in anticipation of an upcoming social event (Brozovich & Heimberg, 2008) [21]. Self-focused attention and negative beliefs and assumptions were found to mediate the SA and post-event processing. A negative self-image is more likely to result in less positive post-event processing [17-20]. The cognitive-behavior model points out attentional bias away from positive aspects of self, others, and the situation coupled with dysfunctional emotional regulation leading to negative affect leads to fear of social events. This fear is aggravated by adapting dysfunctional self-protective behavior and maintained by negative post-event evaluation.

## 3 METHODOLOGY

### 3.1 Participants and Procedure

Data were obtained using a convenient sample of college students in Chennai city in Southern India. Chennai is one of the major metropolitan cities in India and has a culturally diverse population. We collected data from eight arts and science colleges across all four city zones (North, East, West, and Central). After obtaining permission from the head of the institution of the colleges, we distributed flyers of the study on the college notice board and social media networks involving students. Interested participants approached the research assistants, and they were briefed on the research study.

After ascertaining their willingness to participate in the research study, informed consent was taken from the participants, and they were given the survey forms; a total of 905 students filled out the survey. We found that data from 821 participants were complete, and these data were included in the final analysis. The participants took approximately 20 minutes to complete the survey.

### 3.2 Translation of the Tools and the Pilot- study

Because most of the participants in this study came from a Tamil-speaking region (Tamil Nadu state), we translated the measures into Tamil after obtaining permission from the authors of each measure. An independent bi-lingual expert carried out Tamil translation of all the measures. The Tamil versions were then back-translated by another expert. A committee of experts involving the bi-lingual experts and the research team discussed the translations and approved the Tamil versions after suggesting a few modifications. The translated version of the measures was administered to 50 college students for pilot- testing. The participants offered minor suggestions and took approximately 20 minutes to complete the survey.

### 3.3 Measures

Translated and pilot-tested versions of the following questionnaires were administered:

1. A short form of the Self-Compassion Scale (SCS) [38, 39] containing 12 statements to be responded to on a 5-point rating scale (1=almost never to 5=almost always). The Cronbach's alpha value of the SCS in the current study was 0.84.

2. Positive and negative affect scale [5] containing 20 adjectives, ten adjectives for describing each effect, to be responded to on a 5-point rating scale (1=very slight or not at all to 5=extremely). The Cronbach's alpha value of the PANAS in the current study was 0.79 (positive) and .082 (negative).

3. Five-facet mindfulness questionnaire [40] consisting of 15 statements to be responded to on a 5-point scale (1=Never or very rarely true to 5=very often or always true). The Cronbach's alpha value of the FFMQ in the current study was 0.82.

4. Liebowitz social anxiety scale [41] consists of 24 social situations to be responded to twice – once for social fear and once for social avoidance- on a 4-point response scale (0=none to 3=severe). The Cronbach's alpha value of the LSAS in the current study was 0.84.

#### 3.3.1 Hypotheses

Many previous studies have shown the relationships among the variables of the study. However, with specific insights from studies linking social anxiety with self-compassion [25], positive and negative affect, and mindfulness [24, 32, 33], the following hypotheses were formulated for the present study:

• Hy1: Increase in Self-compassion increases mindfulness.

• HY2: Increase in Positive affect increases mindfulness.

• Hy3: An increase in Negative affect decreases mindfulness.

• Hy4: An increase in Mindfulness decreases social fear.

• Hy5: An increase in Social fear increases social avoidance.

• Hy6: Mindfulness mediates the relationship between self-compassion, positive affect, and negative affect on the one hand and social fear on the other.

• Hy7: Mindfulness mediates the relationship between self-compassion, positive affect, and negative affect on the one hand and social avoidance on the other.

• Hy8: Mindfulness and social fear jointly mediate the relationship between self-compassion, positive affect, and negative affect on the one hand and social avoidance on the other.

• Hy9: Mindfulness and social fear jointly mediate the relationship between self-compassion, positive affect, and negative affect on the one hand and social avoidance on the other.

#### 3.3.2 The Measurement Models

Responses were scored to generate metrics for five variables, namely, self-compassion (SC), positive affect (PA), negative affect (NA), mindfulness (MD), social fear (SFR), and social avoidance (SAV). The whole scale and its components were examined for normality and linearity. Table **1** displays the relevant statistics for summated item scores for each variable. The skewness and kurtosis values are within ± 1 range for all variables but for mindfulness, which has a slight negative skewness. Intercorrelation between variables is significant and linear. Factor structures of individual scales were examined, and item parcels were generated for further analysis. Within each subscale of each questionnaire, items were randomly grouped into parcels.

Exploratory factor analysis (EFA) was performed on item parcels. Table **2** displays the factor loadings of each item parcel with the respective factor. Factor extraction was done using the principal component method with varimax rotation. Six orthogonal factors were extracted. Each item parcel is satisfactorily explained by all the six factors as shown by communality (h^2^) values, which range from 0.683 to 0.852. Put together, 75.435% of the variance in all 15 item parcels has been accounted for. Each set of item parcels loaded high in one factor and very low in others, suggesting excellent convergence.

To rule out the possibility of the common method bias, generally found in self-report measures, all item parcels were loaded on a single factor, and factor extraction was done without rotation. 31.14% of the variance has been explained from all item parcels. This is less than 50%, ruling out the possibility of method-induced variance (Herman, 1960; Podsakoff *et al*., 2003) [35, 36].

To confirm the factor structure unearthed by EFA and to estimate the convergent and discriminant validity of latent constructs, confirmatory factor analysis (CFA) was performed on item parcels. Table **3** and Fig. (**2**) display the factor loading of CFA.

The factor loadings in magnitude and direction look similar to the loadings estimated by EFA. There is sufficient convergence of item parcels with their respective latent factors but for self-compassion. Each latent factor is sufficiently distinct from the other. Table **4** presents the convergent and discriminant validity of the latent factors. Though the convergence of items in the self-compassion scale is lower than the expected level (0.5 is the threshold level), it is sufficiently distinct from other factors. It, hence, can be treated as a distinct construct.

### 3.4 Analytical Strategy

The conceptual model in Fig. (**1**) was fitted to observed data using a structural equation model (SEM) with multiple mediation effects using AMOS ver. 24. Direct effects of exogenous construct SC, PA, and NA on the endogenous constructs SFR and SAV, before and after introducing mediating construct MD, were compared to estimate the type and magnitude of mediation effects. For estimating multiple mediations, both MD and SFR were used as mediators.

## 4 RESULTS

Structural path coefficients are displayed in Table **5** and Fig. (**3**). All model fit indices suggest a good fit. The Goodness of Fit (GFI) is 0.979, the Comparative fit index (CFI) is 0.987, and the Root mean square error of approximation (RMSEA) is 0.031. All direct and indirect path coefficients were estimated to arrive at mediation effects. The unstandardized and standardized path coefficients are presented in Table **5**. Fig. (**3**) gives the estimated model. The exogenous constructs SC (0.426; p=0.000) and PA (0.563; p=0.000) positively impact MD, the endogenous mediating construct, and the exogenous construct NA (-0.365; p=0.000) negatively impact MD. The MD (-0.177; p=0.000) negatively impacts the mediating construct SFR; SFR positively impacts the endogenous construct SAV. All the hypotheses are accepted, and the overall model fits the conceptual logic propounded by the study.

### 4.1 Mediation Effects

The basic model being a mediation model, the magnitude and nature of the mediation by MD and SFR individually and jointly must be estimated to gain a clearer understanding. All paths, both direct and indirect, must be estimated. Table **5** shows the relevant path coefficients. The mediation effects were estimated by comparing the direct path coefficients of exogenous constructs to endogenous constructs before and after introducing mediator constructs. The indirect effect (mediation effect) and the change in the direct path coefficients before and after introducing mediators were examined to assess the magnitude and the nature of mediation. Table **6** presents the path coefficients before and after mediation and the mediation effects of both MD and SFR individually and jointly.

Fig. (**4a**) depicts the mediating relationship of SC on SFR and SAV. MD mediates the impact of SC on SFR. Before introducing MD, the effect of SC on SFR is significant (-0.128; p=0.000), and after introducing MD, this effect reduces to insignificance (-0.053; p=0.226). The mediating construct MD explains a substantial portion of this effect (-0.075; p=0.000). Self-compassion reduces social fear by increasing mindfulness.

MD does not mediate the impact of SC on SAV. The direct paths before (-0.021; p=0.570) and after (-0.038; p=0.385) mediation do not change, and the mediation effect (0.017; p=0.394) is also insignificant. Interestingly, the joint mediation of MD and SFR on the impact of SC on SAV is significant (-0.031; p=0.000) but not sufficient to effect a significant change in the path coefficients before (-0.070; p=0.124) and after (-0.038; p=0.385) joint mediation.

Fig. (**4b**) depicts the mediating effects of PA on SFR and SAV. The mediating effects are very similar to that of SC on SFR and SAV. MD mediates the effect of PA on SFR (-0.099; p=0.000). The change in path coefficients of PA on SFR before (-0.107; p=0.001) and after (-0.008; p=0.853) mediation indicates full mediation. The mediation effect of MD on the impact of PA on SAV is not significant (0.022; p=0.400), which is also reflected in the absence of substantial change in path coefficients before (0.015; p=0.663) and after (-0.007; p=0.821) mediation. The joint mediation of MD and SFR is significant (-0.042; p=0.000) but not sufficient to produce substantial change in path coefficients before (-0.048; p=0.254) and after (-0.007; p=0.821).

Fig. (**4c**) displays the mediating effects of NA on SFR and SAV. MD partially mediates the impact of NA on SFR. The mediating effect (0.064; p=0.000) is significant, but the path coefficients from before (0.192; p=0.000) to after (0.127; p=0.001) are reduced but not substantial. Though NA on its own can increase social fear, with MD as a mediator, this debilitating effect is attenuated to a substantial degree.

NA does not impact SAV either directly (0.000; *p* =.992) or through MD (0.014; p=0.635), which is reinforced by the insignificant mediating effect (-0.014; p=0.404). Both MD and SFR have jointly attenuated the impact of NA on SAV (0.027; p=0.000), but this is not substantial enough to effect any meaningful change in the path coefficients before (0.041; p=0.190) and after (0.014; p=0.635) joint mediation.

## 5 DISCUSSION

As presumed in this study, the components of social anxiety-social fear and social avoidance- emerge as two distinct constructs. Hence, summing these components as a single measure of social anxiety could confound its interplay with other constructs. Good convergent and discriminant validity of these two components warrant their treatment as two separate constructs influencing each other. Fear of social situations would lead to avoidance of social situations. This defensive avoidance of social situations would preclude the opportunities to learn social skills. This would operate as a vicious cycle and deprive the individuals of developing social competencies. Female college students, the target population of this study, could easily get into this vicious cycle and develop social incompetency and, therefore, become victims of unfair social treatment. A better understanding of antecedent factors controlling the fear of social situations is essential to deal with this unfortunate social malice.

The results of the study supported the model that self-compassion and emotional well-being influence mindfulness, and mindfulness attenuates social fear, and reduced social anxiety reduces the tendency towards social avoidance. The path coefficients depicting these relationships are all in the predicted magnitude and direction. The notable aspect of the model is the mediating influence of mindfulness. Being compassionate to oneself with a positive emotionality does not directly influence social fear or avoidance. They perhaps predispose individuals to accept and acknowledge themselves and help to create a clearer perspective of their probable reactions to the social situation and its ramifications. Looking at the social situation with a clearer perspective would help them ward off the fear emanating out of uncertainty of the situation and the lack of alternatives in dealing with it. Reduced fear would encourage the individual to approach the social situation with much preparedness and openness. Though it was not a part of the presumed model, mediating effects of mindfulness and social fear on social anxiety were estimated.

Social fear appears to be a crucial variable responsible for social avoidance. To reduce social avoidance and thus enhance social participation, reducing fear of social situations is necessary. Two counterbalancing forces appear to have a strong impact on social fear. Mindfulness, which is the awareness and acceptance of the feelings, thoughts, and sensations attached to self and its possible reciprocity with social surroundings, seeks to mitigate the fear and negative emotional effects to enhance the fear. Mindfulness could be nurtured through training to increase self-compassion and positive emotionality and decrease negative emotionality. Decreasing negative emotionality appears to have a more substantial impact on social fear through increasing mindfulness, thereby reducing fear and directly mitigating fear. These inferences are very relevant to the population of the study: urban, middle-class young women of college-going age growing up in relatively traditional cultural settings. However, the universality of these inferences is to be tested across various geographical locations and cultural contexts.

## CONCLUSION

1. The proposed mediating model holds for the data. The dispositional variables, namely, self-compassion and positive emotional affect, augment and negative emotional affect, attenuate mindfulness. Mindfulness weakens social fear, and social fear reduces the tendency to avoid social situations.

2. Mindfulness effectively mediates the impact of self-compassion's positive effects and negative effects on social fear. The extent of covariance accounted for by mindfulness is substantial. While mindfulness fully mediates with respect to self-compassion and positive affect, it partially mediates the negative effect.

3. Mindfulness does not mediate the impact of self-compassion's positive and negative effects on social avoidance.

4. Mindfulness and social fear jointly mediate the impact of self-compassion, positive affect, and negative affect on social avoidance. The mediation effect is rather weak as it does not substantially change the impact before and after introducing mediators.

## LIMITATIONS AND FUTURE DIRECTIONS

Our study has certain limitations. First, although we have used robust statistical analyses, the nature of the data is cross-sectional in nature. This would limit causal inference of the results of the study. We have used urban and educated college students as participants. The generalization of the findings to other demographic groups needs careful consideration. The measures translated in this study can be validated in future cross-cultural studies.

## Figures and Tables

**Fig. (1):**
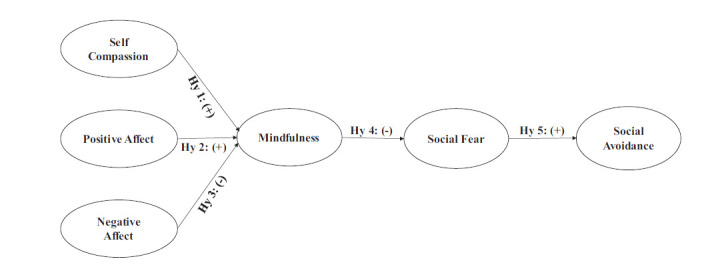
Conceptual model.

**Fig. (2):**
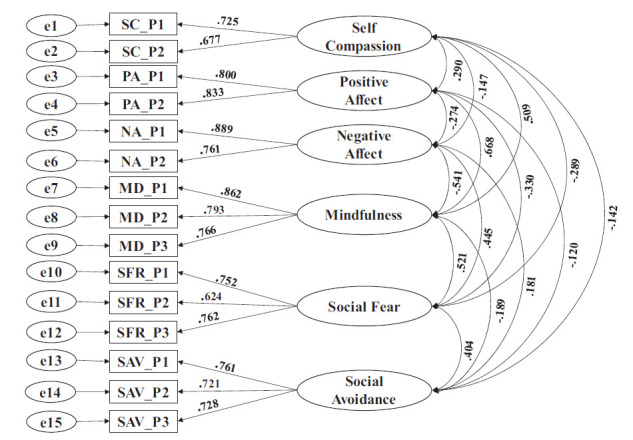
Measurement model.

**Fig. (3):**
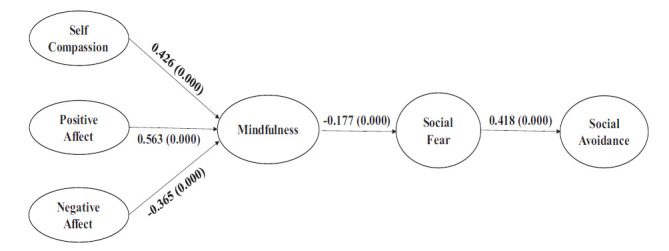
Estimated model.

**Fig. (4a):**
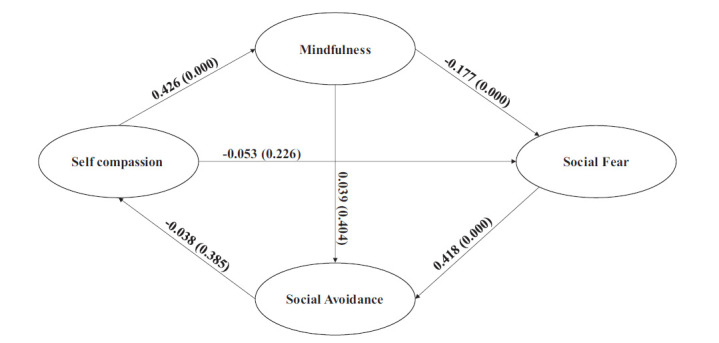
Mediation Effects of Mindfulness and Social Fear (Self Compassion → Social Avoidance).

**Fig. (4b):**
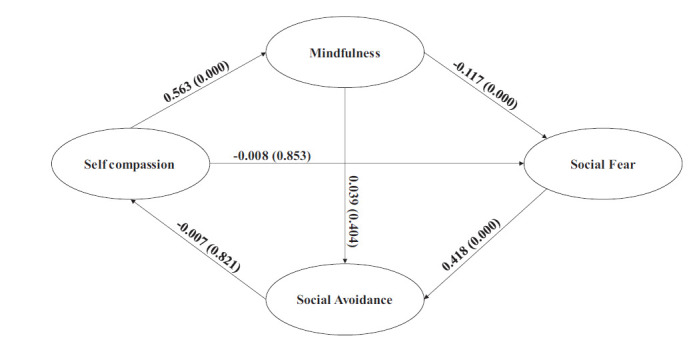
Mediation Effects of Mindfulness and Social Fear (Positive Affect → Social Avoidance).

**Fig. (4c):**
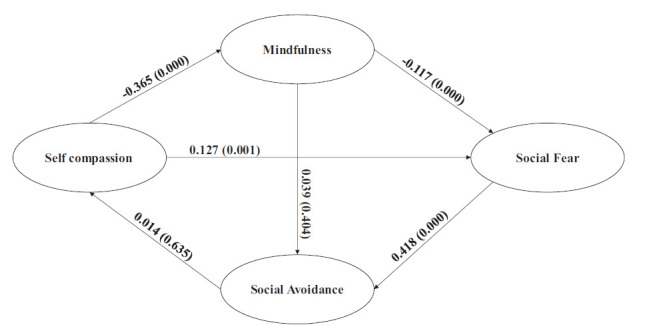
Mediation Effects of Mindfulness and Social Fear (Negative affect → Social Avoidance).

**Table 1:** Descriptive statistics and intercorrelations of variables.

Variables	Descriptives				Inter-correlations					
	Mean	Std. Deviation	Skewness	kurtosis	SC	PA	NA	MD	SFR	SAV
Self-compassion (SC)	29.569	6.714	-0.062	-0.724	1	0.206**	-0.100**	0.391**	-0.196**	-0.098**
Positive affect (PA)	32.540	7.332	-0.243	-0.632	-	1	-0.214**	0.554**	-0.260**	-0.093**
Negative affect (NA)	24.792	8.271	0.087	-0.792	-	-	1	-0.440**	0.354**	0.136**
Mindfulness (MD)	45.921	10.699	-1.202	0.989	-	-	-	1	-0.418**	-0.148**
Social Fear (SFR)	17.040	6.689	0.138	-0.340	-	-	-	-	1	0.305**
Social Avoidance	16.582	6.314	-0.102	-0.493	-	-	-	-	-	1

Note: **Significant beyond 0.01 level (*N*=*821*).

**Table 2:** Exploratory factor analysis.

-	Factors						
Item Parcels	1	2	3	4	5	6	h^2^
MD_P2	0.825	-0.081	-0.181	-0.170	0.173	0.108	0.725
MD_P1	0.813	-0.044	-0.156	-0.213	0.258	0.131	0.766
MD_P3	0.713	-0.024	-0.158	-0.143	0.268	0.271	0.686
SAV_P2	0.043	-0.843	0.068	-0.012	-0.036	-0.021	0.687
SAV_P1	-0.121	0.819	0.125	0.035	-0.045	-0.051	0.693
SAV_P3	-0.048	0.806	0.151	0.095	0.015	-0.014	0.707
SFR_P2	-0.034	0.080	0.808	0.102	-0.126	0.026	0.720
SFR_P3	-0.158	0.179	0.780	0.135	-0.071	-0.071	0.683
SFR_P1	-0.257	0.128	0.756	0.106	-0.017	-0.143	0.837
NA_P2	-0.149	0.049	0.154	0.895	-0.044	-0.002	0.828
NA_P1	-0.250	0.064	0.157	0.852	-0.084	-0.041	0.825
PA_P1	0.241	-0.045	-0.108	-0.104	0.868	0.023	0.852
PA_P2	0.284	0.018	-0.090	-0.021	0.851	0.118	0.815
SC_P2	0.119	-0.029	-0.062	-0.074	0.058	0.859	0.790
SC_P1	0.200	-0.047	-0.067	0.040	0.064	0.820	0.700
Eigenvalue	2.238	2.107	2.033	1.690	1.689	1.559	11.315
% variance extracted	14.919	14.045	13.554	11.264	11.260	10.392	75.435

**Table 3:** Confirmatory factor analysis.

Indicators	-	Constructs	Unstd.Est.	Std.Error	p	Std.Est.
SC_P1	←	Self-compassion	2.787	0.178	0.000	0.725
SC_P2	←		2.659	0.176	0.000	0.677
PA_P1	←	Positive Affect	3.178	0.137	0.000	0.800
PA_P2	←		3.375	0.140	0.000	0.833
NA_P1	←	Negative Affect	3.742	0.154	0.000	0.889
NA_P2	←		3.665	0.174	0.000	0.761
MD_P1	←	Mindfulness	3.752	0.128	0.000	0.862
MD_P2	←		3.187	0.123	0.000	0.793
MD_P3	←		2.942	0.119	0.000	0.766
SFR_P1	←	Social Fear	1.942	0.089	0.000	0.752
SFR_P2	←		1.490	0.084	0.000	0.624
SFR_P3	←		2.403	0.108	0.000	0.762
SAV_P1	←	Social Avoidance	1.970	0.090	0.000	0.761
SAV_P2	←		2.038	0.098	0.000	0.721
SAV_P3	←		1.556	0.074	0.000	0.728

**Table 4:** Convergent and discriminant validity of constructs.

Constructs	Convergent Validity			Discriminant Validity					
	AVE	CR	α	SC	PA	NA	MD	SFR	SAV
Self-compassion (SC)	0.492	0.659	0.658	**0.492**	0.084	0.022	0.259	0.084	0.020
Positive Affect (PA)	0.667	0.800	0.799	-	**0.667**	0.075	0.446	0.109	0.014
Negative Affect (NA)	0.685	0.812	0.803	-	-	**0.685**	0.293	0.198	0.033
Mindfulness (MD)	0.653	0.849	0.846	-	-	-	**0.653**	0.271	0.036
Social Fear (SF)	0.713	0.757	0.751	-	-	-	-	**0.713**	0.163
Social Avoidance (SAV)	0.543	0.781	0.774	-	-	-	-	-	**0.543**

**Note: ***AVE* = *Average variance extracted; CR* = *Composite reliabilityα* = *Cronbach's reliability coefficients estimated for item parcelsUnder discriminant validity, the diagonals represent AVEs, and off-diagonal elements are squared correlations*.

**Table 5:** Structural path coefficients.

Constructs			Unstd.Est.	Std.Error	Critical Ratio	p	Std.Est.
Mindfulness	←	Self-compassion	0.426	0.057	7.512	0.000	0.317
Mindfulness	←	Positive Affect	0.563	0.045	12.380	0.000	0.477
Mindfulness	←	Negative Affect	-0.365	0.036	-10.028	0.000	-0.364
Social Fear	←	Self-compassion	-0.053	0.040	-1.324	0.186	-0.076
Social Fear	←	Positive Affect	-0.008	0.038	-0.208	0.835	-0.013
Social Fear	←	Negative Affect	0.127	0.028	4.481	0.000	0.245
Social Avoidance	←	Self-compassion	-0.038	0.043	-0.880	0.379	-0.054
Social Avoidance	←	Positive Affect	-0.007	0.041	-0.164	0.870	-0.011
Social Avoidance	←	Negative Affect	0.014	0.030	0.474	0.636	0.027
Social Fear	←	Mindfulness	-0.177	0.046	-3.882	0.000	-0.0341
Social Avoidance	←	Mindfulness	0.039	0.050	0.790	0.429	0.075
Social Avoidance	←	Social Fear	0.418	0.060	6.948	0.000	0.412

**Note: ***Model fit: Model chi-sq=133.951 (df*=*75); GFI* = *0.979; CFI* = *0.987; RMSEA* = *0.031*.

**Table 6:** Mediation effects.

Path Nos	Paths							Before Mediation		Mediation		After Mediation		Nature of Mediation
								B	p	B	p	B	p	
1	-	-	SC	→	MD	→	SFR	-0.128	0.001	-0.075	0.000	-0.053	0.226	Full mediation
2	-	-	SC	→	MD	→	SAV	-0.021	0.570	0.017	0.394	-0.038	0.385	No mediation
3	-	-	SC	→	SFR	→	SAV	-0.06	0.214	-0.022	0.221	-0.038	0.385	No mediation
4	SC	→	MD	→	SFR	→	SAV	-0.070	0.124	-0.031	0.000	-0.038	0.385	Weak mediation
5	-	-	PA	→	MD	→	SFR	-0.107	0.001	-0.099	0.000	-0.008	0.853	Full mediation
6	-	-	PA	→	MD	→	SAV	0.015	0.663	-0.022	0.400	-0.007	0.821	No mediation
7	-	-	PA	→	SFR	→	SAV	-0.01	0.738	-0.003	0.855	-0.007	0.821	No mediation
8	PA	→	MD	→	SFR	→	SAV	-0.048	0.254	-0.042	0.000	-0.007	0.821	Weak mediation
9	-	-	NA	→	MD	→	SFR	0.192	0.000	0.064	0.000	0.127	0.001	Partial mediation
10	-	-	NA	→	MD	→	SAV	0.000	0.992	-0.014	0.404	0.014	0.635	No mediation
11	-	-	NA	→	SFR	→	SAV	0.068	0.033	0.053	0.000	0.014	0.635	Full mediation
12	NA	→	MD	→	SFR	→	SAV	0.041	0.190	0.027	0.000	0.014	0.635	Weak mediation

## Data Availability

The data and the supporting information are available from the corresponding author [B.K] on a reasonable request.

## LIST OF ABBREVIATIONS

SAD: Social Anxiety Disorder
SC: Self-compassion
PA: Positive emotional affect
NA: Negative emotional affect
